# Thermal Detection Thresholds of Aδ- and C-Fibre Afferents Activated by Brief CO_2_ Laser Pulses Applied onto the Human Hairy Skin

**DOI:** 10.1371/journal.pone.0035817

**Published:** 2012-04-25

**Authors:** Maxim Churyukanov, Léon Plaghki, Valéry Legrain, André Mouraux

**Affiliations:** 1 Institute of Neuroscience (IONS), Université catholique de Louvain, Brussels, Belgium; 2 Department of Nervous Diseases, The I.M. Sechenov First Moscow State Medical University, Moscow, Russia; 3 Laboratory of Pathophysiology of Pain, Institute of General Pathology and Pathophysiology RAMS, Moscow, Russia; 4 Department of Experimental Clinical and Health Psychology, Ghent University, Ghent, Belgium; University of Manchester, United Kingdom

## Abstract

Brief high-power laser pulses applied onto the hairy skin of the distal end of a limb generate a double sensation related to the activation of Aδ- and C-fibres, referred to as first and second pain. However, neurophysiological and behavioural responses related to the activation of C-fibres can be studied reliably only if the concomitant activation of Aδ-fibres is avoided. Here, using a novel CO_2_ laser stimulator able to deliver constant-temperature heat pulses through a feedback regulation of laser power by an online measurement of skin temperature at target site, combined with an adaptive staircase algorithm using reaction-time to distinguish between responses triggered by Aδ- and C-fibre input, we show that it is possible to estimate robustly and independently the thermal detection thresholds of Aδ-fibres (46.9±1.7°C) and C-fibres (39.8±1.7°C). Furthermore, we show that both thresholds are dependent on the skin temperature preceding and/or surrounding the test stimulus, indicating that the Aδ- and C-fibre afferents triggering the behavioural responses to brief laser pulses behave, at least partially, as detectors of a change in skin temperature rather than as pure level detectors. Most importantly, our results show that the difference in threshold between Aδ- and C-fibre afferents activated by brief laser pulses can be exploited to activate C-fibres selectively and reliably, provided that the rise in skin temperature generated by the laser stimulator is well-controlled. Our approach could constitute a tool to explore, in humans, the physiological and pathophysiological mechanisms involved in processing C- and Aδ-fibre input, respectively.

## Introduction

For the past 30 years, investigators have relied extensively on infrared laser stimulators to study nociception and pain perception in humans [1]–[4]. Indeed, as a source of radiant heat applied onto the skin, infrared lasers can be used to activate heat-sensitive Aδ- and C-fibre afferents selectively. Most importantly, because of their very high power output, infrared lasers can generate the very steep heating ramps required to elicit time-locked responses such as reaction-times and event-related brain potentials [5], and are thus well suited to study the human nociceptive system.

However, for reasons that remain a matter of debate, brain responses related to the activation of C-fibres can be identified only if the concomitant activation of Aδ-fibres is avoided [6]–[8]. Therefore, in humans, the current research has focussed almost exclusively on characterizing the neural processes triggered by Aδ-fibre input, simply because of the lack of reliable methods to study the responses triggered by the selective activation of C-fibres. Hence, to progress in our understanding of the physiology and pathophysiology of nociception, developing means to study the signals ascending through C-fibres is essential [9].

Based on characteristics differentiating Aδ- and C-fibres, several methods have been proposed to activate C-fibre afferents selectively [5]. A first method exploits the fact that unmyelinated C-fibres are more resistant to pressure than myelinated A-fibres, and consists in applying prolonged force against a peripheral nerve such as to block selectively the nerve conduction of A-fibres [10], [11]. A second method takes advantage of the fact that the distribution density of C-fibres in the epidermis is greater than that of Aδ-fibres, and consists in using a very small stimulation surface area to elicit isolated C-fibre responses [12], [13]. Indeed, Ochoa et al. [14] estimated a 1/4 ratio between Aδ- and C-fibres in a human peripheral sensory nerve, suggesting that the intraepidermal density of C-fibres is greater than that of Aδ-fibres. However, because of the lack of histological staining methods to differentiate between Aδ- and C-fibre free nerve endings in the epidermis, their actual respective distribution remains to be established. A third method uses the difference in the thermal activation thresholds of Aδ- and C-fibre afferents, and consists in heating the skin above the threshold of C-fibres, but below the threshold of Aδ-fibres [15]. However, all of these methods are technically challenging to implement, and the results they yield are often unreliable. Hence, at present, their use as a routine clinical diagnostic tool is not conceivable [9].

Here, we propose the basis for a robust approach to estimate the thermal detection thresholds of heat-sensitive Aδ- and C-fibre afferents, using a new CO_2_ laser stimulator able to deliver constant-temperature heat pulses through a feedback regulation of laser power by an online measurement of skin temperature at target site, combined with an adaptive psychophysical algorithm using reaction-times as criterion to distinguish between responses triggered by Aδ- and C-fibre input. Furthermore, we show that the difference between C- and Aδ-fibre thresholds can be exploited to activate C-fibre afferents selectively and reliably, provided that the temperature of the eliciting stimulus is well controlled. By providing a unique mean to characterize the neural activity triggered by C-fibre input in humans, for example, through the recording of ultra-late C-fibre event-related brain potentials, our approach could constitute an interesting tool to study the contribution of C-fibres to both physiological and pathological pain.

## Methods

### Participants

Nine healthy participants (3 females; 8 right-handed, aged 24–35 years) took part in the study. Before the experiment, they were familiarized with the experimental setup and task. Experimental procedures were approved by the Ethics Committee of the Université catholique de Louvain (B40320096449). Written informed consent was obtained from participants.

### CO_2_ laser stimulation of Aδ- and C-fibre afferents

**Figure 1:**
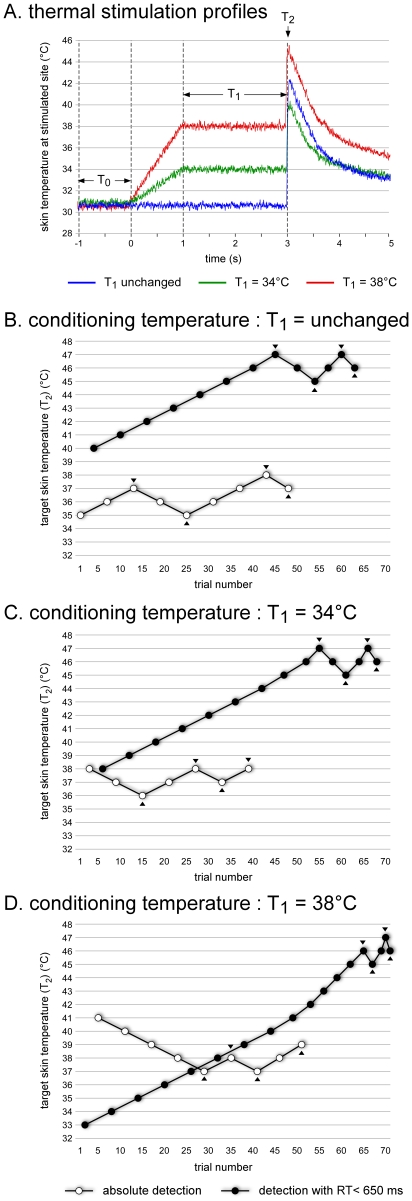
Experimental paradigm. A. Thermal stimuli were applied to the dorsum of the non-dominant hand using a CO_2_ laser (beam diameter: 6 mm). The data shown corresponds to an actual measurement of skin temperature performed by the radiometer in three different trials (x-axis: time; y-axis: skin temperature). After measuring the baseline skin temperature for one second (T_0_), the stimulated area was raised to the conditioning skin temperature using a 1-s heating ramp and was maintained at that temperature for 2 seconds (T_1_). Three different conditioning skin temperatures were used: T_1_ = unchanged (blue), T_1_ = 34°C (green) and (c) T_1_ = 38°C (red). Following this conditioning of the stimulated area, the test stimulus (T_2_) was applied, consisting of a 10-ms heating ramp followed by a 40-ms plateau. B–D: The staircase procedure used to estimate Aδ- and C-fibre thresholds (one subject shown as example). Participants were asked to respond as quickly as possible by pressing a button held in the dominant hand when perceiving the test stimulus (T_2_). Reaction times were used to discriminate between responses triggered by C-fibre input (reaction-time ≥650 ms) and responses triggered by Aδ-fibre input (reaction-time <650 ms). For all three conditions (Panel B: T_1_ = unchanged, Panel C: T_1_ = 34°C and Panel D: T_1_ = 38°C), an adaptive staircase algorithm was used to estimate (1) the absolute detection threshold assumed to reflect the detection threshold of C-fibre input and (2) the detection threshold of responses with a reaction-time <650 ms assumed to reflect the detection threshold of Aδ-fibre input. The six staircases required for this procedure were presented in an interleaved fashion, such as to avoid order effects related to habituation and/or sensitization as well as a possible response bias due to anticipation.

Thermal stimuli were applied to the dorsum of the non-dominant hand, using a new CO_2_ laser stimulator whose power is regulated using a feedback control based on an online measurement of skin temperature at the site of stimulation (Laser Stimulation Device, SIFEC, Belgium). The device is commercially available, and approved for medical use. Conception of the laser was inspired by the temperature-controlled laser stimulator proposed by Meyer et al. [16]. Both devices are based on a closed-loop control of laser power by an online monitoring of skin temperature performed using a radiometer collinear with the laser beam. As compared to the device proposed by Meyer et al. [16], the present device integrates some improvements provided by recent technical progress. The most important difference is the very small lag in the feedback control. Therefore, by sampling the fast-adapting output of the radiometer at a rate of 500 Hz, it is possible to achieve temperature steps with much greater rise rates. For example, in Magerl et al. [15], heating ramps were approximately 50°C/s and, consequently, the time required to bring the skin temperature from baseline to a temperature supraliminal for Aδ-nociceptors was approximately 150 ms. In contrast, the present stimulator is able to reach similar target temperatures in less than 10 ms, and is thus better suited to record and interpret time-locked responses such as reaction-times and event-related potentials [17]. The heat source is a 25 W radio-frequency excited C0_2_ laser (Synrad 48-2; Synrad, USA). Power control is achieved by pulse width modulation (PWM) at 5 KHz clock frequency. Stimuli are delivered through a 10 m optical fibre. By vibrating this fibre at some distance from the source, a quasi-uniform spatial distribution of radiative power within the stimulated area is obtained. At the end of the fibre, optics collimate the beam, resulting in a 6-mm beam diameter at target site. Using this system, thermal stimulation profiles were defined as follows (Figure 1A).

#### Baseline skin temperature (T_0_)

Prior to the actual stimulus, the skin temperature was measured for 1000 ms without delivering any laser energy, such as to obtain an estimate of the baseline skin temperature (average of 500 successive measures of skin temperature, sampled at a rate of 500 Hz).

#### Conditioning skin temperature (T_1_)

To examine whether Aδ- and C-fibre thermal detection thresholds were dependent on the skin temperature immediately preceding the stimulus, three different conditioning temperatures were used: *unchanged*, *34°C*, and *38°C*. In the *unchanged* condition, no laser energy was delivered during this 3-second time-interval and the skin temperature of the stimulated area was thus unchanged. In the other two conditions, the skin temperature of the stimulated area was raised to either 34°C or 38°C using a 1-second linear heating-ramp. This conditioning temperature was maintained for 2 seconds before applying the target stimulus.

#### Target skin temperature (T_2_)

The skin temperature of the stimulated area was raised briefly to a defined target temperature using a very steep 10-ms heating ramp, and was maintained at that temperature for another 40 ms. As detailed in the next section, possible target skin temperatures ranged from 33°C to 51°C, in steps of 1°C.

Finally, the skin temperature was measured for an additional 2 seconds without delivering any laser energy.

After each trial, the target of the laser was displaced to a random position on the hand dorsum, in order to avoid nociceptor sensitization and/or habituation. The time interval between two consecutive trials varied randomly between 5 and 10 seconds.

### Procedure

Stimuli were delivered and detection thresholds were estimated by means of a staircase algorithm (Figure 1B–D).

**Figure 2:**
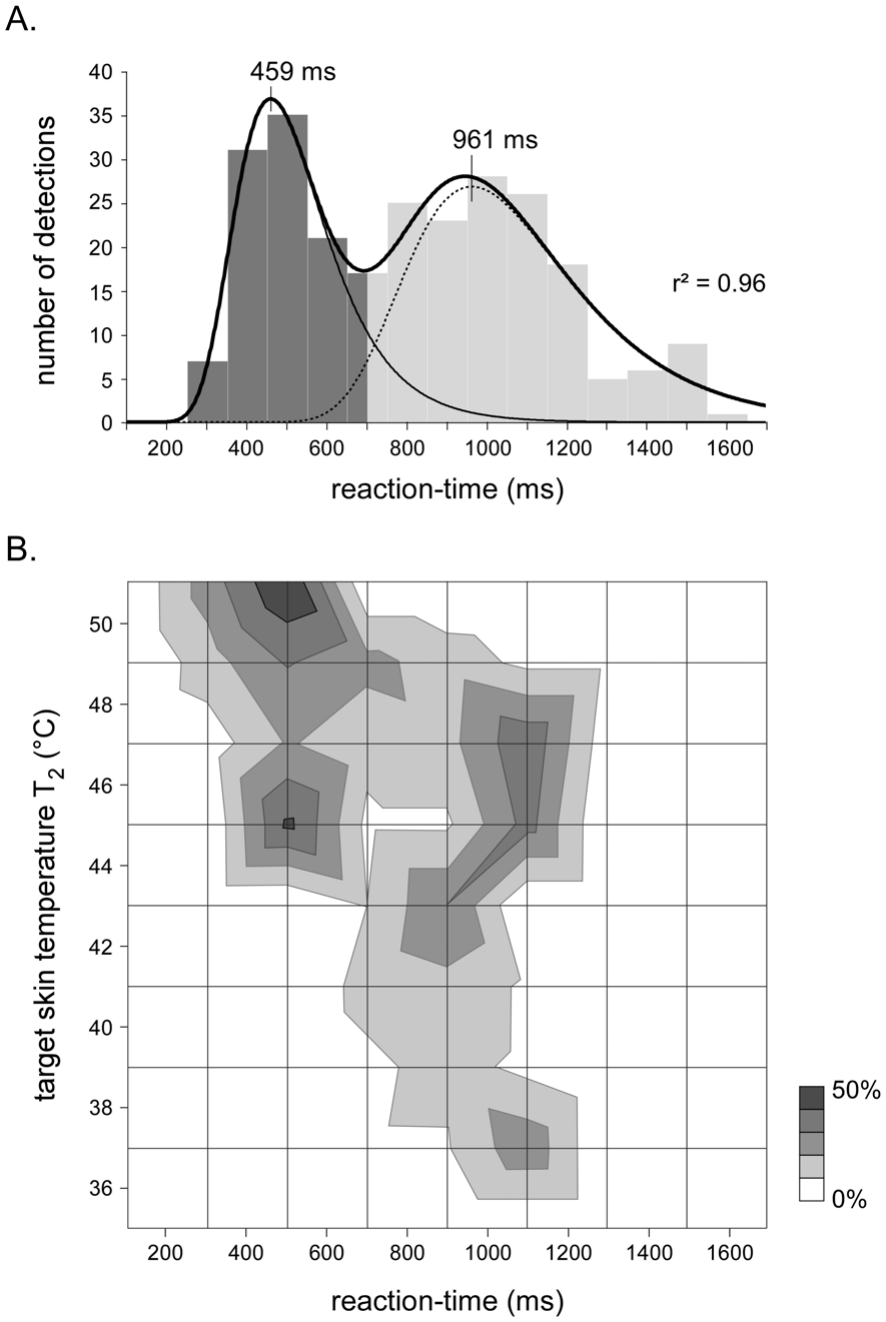
Frequency distribution of the reaction-times to brief CO_2_ laser pulses applied onto the dorsum of the non-dominant hand using target skin temperatures ranging from 35 to 51°C. The upper panel shows the overall distribution of reaction-times regardless of the target skin temperature (T_2_). The lower panel shows the distribution of reaction-times as a function of T_2_. Note the bimodal distribution of reaction-times, related to the fact that lower target skin temperatures triggered late-latency detections compatible with the conduction velocity of unmyelinated C-fibres, whereas higher target skin temperatures triggered early-latency detections compatible with the conduction velocity of myelinated Aδ-fibres (at higher skin temperatures, the stimulus activated both Aδ- and C-fiber afferents, but detections were related to the first-arriving Aδ-fiber afferent volley). The bimodal nature of this distribution was confirmed by comparing the fitting of the data to a model describing a unimodal vs. a bimodal distribution of sensory-motor responses (see Results). The first function of the bimodal model characterized the distribution of the short-latency Aδ-fibre responses (*a* = 459 ms; *b* = 111 ms). The second function characterized the distribution of the late-latency C-fibre responses (*a* = 961 ms; *b* = 202 ms).

Participants were asked to respond as quickly as possibly by pressing a button held in the dominant hand when perceiving the target stimulus (T_2_). Reaction time latencies were used to discriminate between detections triggered by C-fibre input and detections triggered by Aδ-fibre input. This is justified by the fact that the nerve conduction velocity of unmyelinated C-fibres is much slower than the nerve conduction velocity of myelinated Aδ-fibres (±1 m/s vs. ±10 m/s; [8], [11], [18]–[20]). Taking into account the peripheral conduction distance of afferent input originating from the hand, and taking into account the distribution of reaction times to laser stimuli after blocking the conduction of myelinated fibres [10], [11], a criterion of 650 ms was chosen to discriminate between C-fibre responses (reaction time ≥650 ms) and Aδ-fibre responses (reaction time <650 ms) [11], [20]. Additional evidence that reaction-times can be used to distinguish between Aδ- and C-fiber responses is provided by Opsommer et al. [21], showing that the time interval between the two peaks of the bimodal distribution of reaction-times increases with peripheral distance. As shown in Figure 2, this criterion effectively discriminated the two response categories.

Because the thermal activation threshold of C-fibres is known to be lower than the thermal activation threshold of Aδ-fibres [11], [18], it was expected that the skin temperature required to elicit Aδ-fibre responses would be higher than the skin temperature required to elicit C-fibre responses.

#### C-fibre thermal detection threshold

A staircase using detection vs. no detection as criterion, regardless of reaction-time, was used to determine the absolute detection threshold. This threshold is expected to reflect the C-fibre threshold, provided that the C-fibre threshold is lower than the Aδ-fibre threshold.

#### Aδ-fibre thermal detection threshold

A staircase using detection with a reaction-time <650 ms as criterion was used to determine the Aδ-fibre thermal detection threshold. Indeed, as shown in Figure 2, such reaction-times are compatible only with the greater conduction velocity of myelinated Aδ-fibres.

For both staircase algorithms, the temperature of the first stimulus of the staircase was set randomly to a value ranging between 32 and 41°C. The temperature of each of the following test stimuli was determined by whether the participant's response to the preceding stimulus met the defined detection criterion. If the preceding stimulus was detected, the temperature of the upcoming stimulus was decreased by 1°C; else, it was increased by 1°C. Hence, the staircase converged towards the skin temperature at which the probability of detecting the stimulus with the defined criterion was 50%.

Aδ- and C-fibre thresholds were estimated for each of the three conditions (T_1_ = unchanged, T_1_ = 34°C, and T_1_ = 38°C). As shown in the right panel of Figure 1, the six staircases required to estimate the two thresholds for each of the three conditions were presented in an interleaved fashion. This procedure avoided any order effect on the estimated thresholds that could have been induced by habituation and/or sensitization if the staircases had been presented in separate successive blocks. Furthermore, the interleaving procedure ensured that participants were unable to predict the temperature of the upcoming stimulus, and unable to understand the relationship between their response and the temperature of the upcoming stimuli, thus preventing any response bias by anticipation.

Each staircase was interrupted after the occurrence of four staircase reversals (i.e. when the current stimulus was detected and the preceding stimulus undetected, or when the current stimulus was undetected and the preceding stimulus detected). Threshold values were then obtained by averaging the target stimulus temperatures T_2_ at which the four staircase reversals had occurred.

## Results

### Baseline skin temperature

Across subjects, the baseline skin temperature of the hand dorsum, averaged across all trials, ranged from 28.5°C to 33.6°C (31.2±1.7°C, group-level mean ±SD).

### Reaction times

As shown in Figure 2, and as predicted by previous results (e.g. [10]–[12], [21], [22]), the frequency distribution of reaction-times appeared bimodal, and the arbitrarily-defined cut-off to discriminate between Aδ-fibre (reaction-time <650 ms) and C-fibre (reaction-time ≥650 ms) responses effectively separated the two response categories.

The bimodal nature of this distribution was confirmed by comparing directly the fitting of the data to a model describing a unimodal distribution vs. the fitting of the data to a model describing a bimodal distribution. For this purpose, the distribution of reaction-times was modelled using an asymmetrical function proposed by Zaitsev and Skorik [23], shown to describe well the right-skewed distribution of sensory-motor responses:
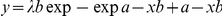



Where *y* is the distribution function of reaction-times *x*, and λ is the width of the class of division. This distribution function is characterized by two parameters: *a* defining the value of *x* at which the distribution function reaches its maximum, and *b* defining the spread of the distribution function.

The bimodal vs. unimodal nature of the distribution of reaction times was then tested by comparing the fitting of the data to this unimodal function (adjusting parameters *a* and *b*), with the fitting of the data to a bimodal model consisting of the sum of these two functions (adjusting parameters *a* and *b* characterizing each of the two functions). The corrected Akaike Information Criterion (AIC) and the F-test were used to select the most likely of the two fitted models. These procedures account for the different number of parameters defining the two models (2 parameters in the unimodal model vs. 4 parameters in the bimodal model). The bimodal model had a lower AIC than the unimodal model (59.2 vs. 89.6). The F-test showed that the data fit significantly better to the bimodal model (r^2^ = 0.96) than to the unimodal model (r^2^ = 0.76) (F = 30.6, p<.0001), thus indicating that the distribution of reaction-times was indeed bimodal. The first function of the bimodal model characterized the distribution of the short-latency Aδ-fibre responses (*a* = 459 ms; *b* = 111 ms). The second function characterized the distribution of the late-latency C-fibre responses (*a* = 961 ms; *b* = 202 ms). Latency of this second peak of the reaction-time distribution was similar to the latencies of the reaction times to laser stimuli obtained after interrupting the conduction of A-fibres using nerve pressure blocks, i.e. in conditions where only the conduction of unmyelinated C-fibres is preserved [10], [11].

### Aδ- and C-fibre thermal detection thresholds

**Figure 3:**
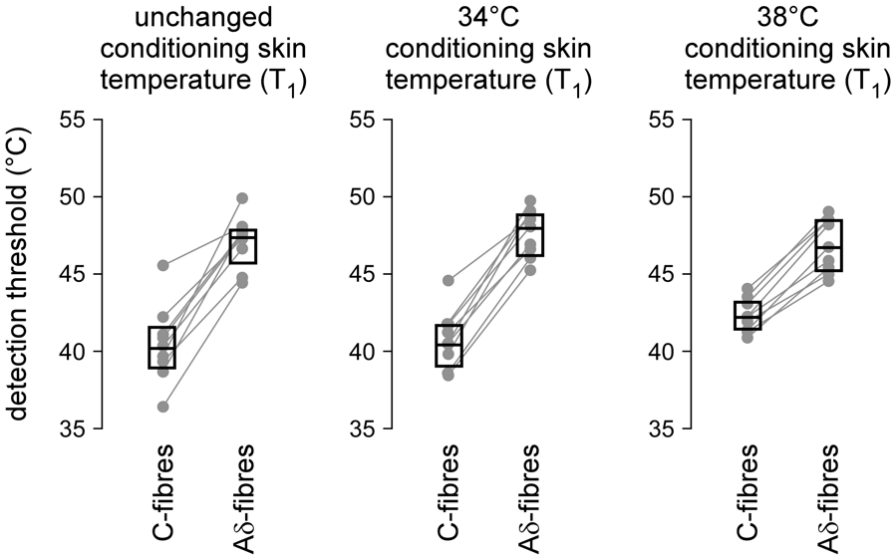
Thermal detection threshold of the behavioural responses triggered by Aδ- and C-fibre input obtained in the three experimental conditions (T_1_ = unchanged, T_1_ = 34°C and T_1_ = 38°C). The thin grey lines represent the individual thresholds obtained in each participant. The box plots represent the group-level median and interquartile range. Note that in all participants, the Aδ-fibre threshold is markedly greater than the C-fibre threshold. Also note that this difference is less marked in the condition T_1_ = 38°C.

**Figure 4:**
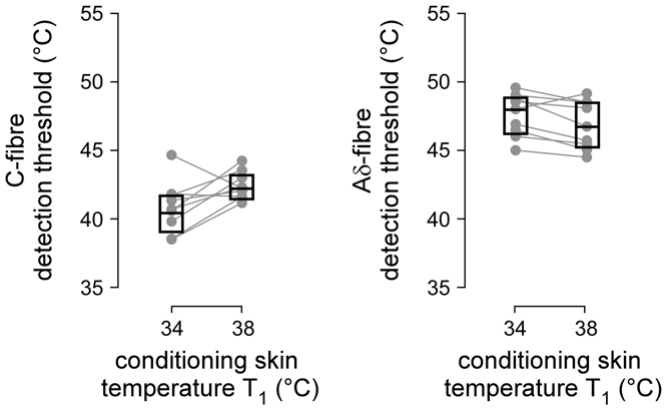
Thermal detection threshold of the behavioural responses triggered by Aδ- and C-fibre input obtained when the stimulated area is first heated to T_1_ = 34°C or T_1_ = 38°C during the two seconds preceding the test stimulus (T_2_). The thin grey lines represent the individual thresholds obtained in each participant. The box plots represent the group-level median and interquartile range. Note that the C-fibre threshold is increased for T_1_ = 38°C vs. T_1_ = 34°C, whereas the Aδ-fibre threshold appears unaffected by the conditioning skin temperature.

When the conditioning skin temperature was unchanged (T_1_ = unchanged), the C-fibre threshold was T_2_ = 39.8±1.7°C, whereas the Aδ-fibre threshold was T_2_ = 46.9±1.7°C (ΔT between C-fibre and Aδ-fibre thresholds: 7.2±1.7°C, ranging from 5.0°C to 10.0°C across participants) (Figure 3). When the conditioning skin temperature was brought to T_1_ = 34°C, the C-threshold was T_2_ = 40.3±1.3°C and the Aδ-threshold was T_2_ = 47.4±1.6°C (ΔT = 7.1±1.4°C, ranging from 5.3°C to 9.3°C). When T_1_ was brought to 38°C, the C-threshold was T_2_ = 42.4±1.0°C and the Aδ-threshold was T_2_ = 46.7±1.8°C (ΔT = 4.3±1.1°C, ranging from 3.0°C to 5.5°C) (Figures 3 and 4).

To assess the difference between Aδ- and C-thresholds, and to examine the effect of the conditioning skin temperature (T_1_), a repeated-measures ANOVA was performed with the following two factors: ‘nociceptor type’ (Aδ or C) and ‘conditioning skin temperature’ (unchanged, 34°C or 38°C). The analysis showed a very significant main effect of the factor ‘nociceptor type’ (F = 481.1, p<.001), a significant main effect of the factor ‘conditioning skin temperature’ (F = 7.5, p = .006), as well as a significant interaction between the two factors (F = 10.8, p = .001). Post-hoc comparisons, performed using the Tukey test, showed that the Aδ-threshold was markedly higher than the C-threshold (ΔT = +6.2°C, p<.001). Furthermore, they showed that the ‘conditioning skin temperature’ significantly affected the C-threshold, which was significantly higher when the conditioning skin temperature was brought to 38°C, as compared to when the conditioning skin temperature was brought to 34°C (ΔT = +2.1°C, p<.001), or when it was unchanged (ΔT = +2.6°C, p<.001). In contrast, the ‘conditioning skin temperature’ had no significant effect on the Aδ-threshold.

**Figure 5:**
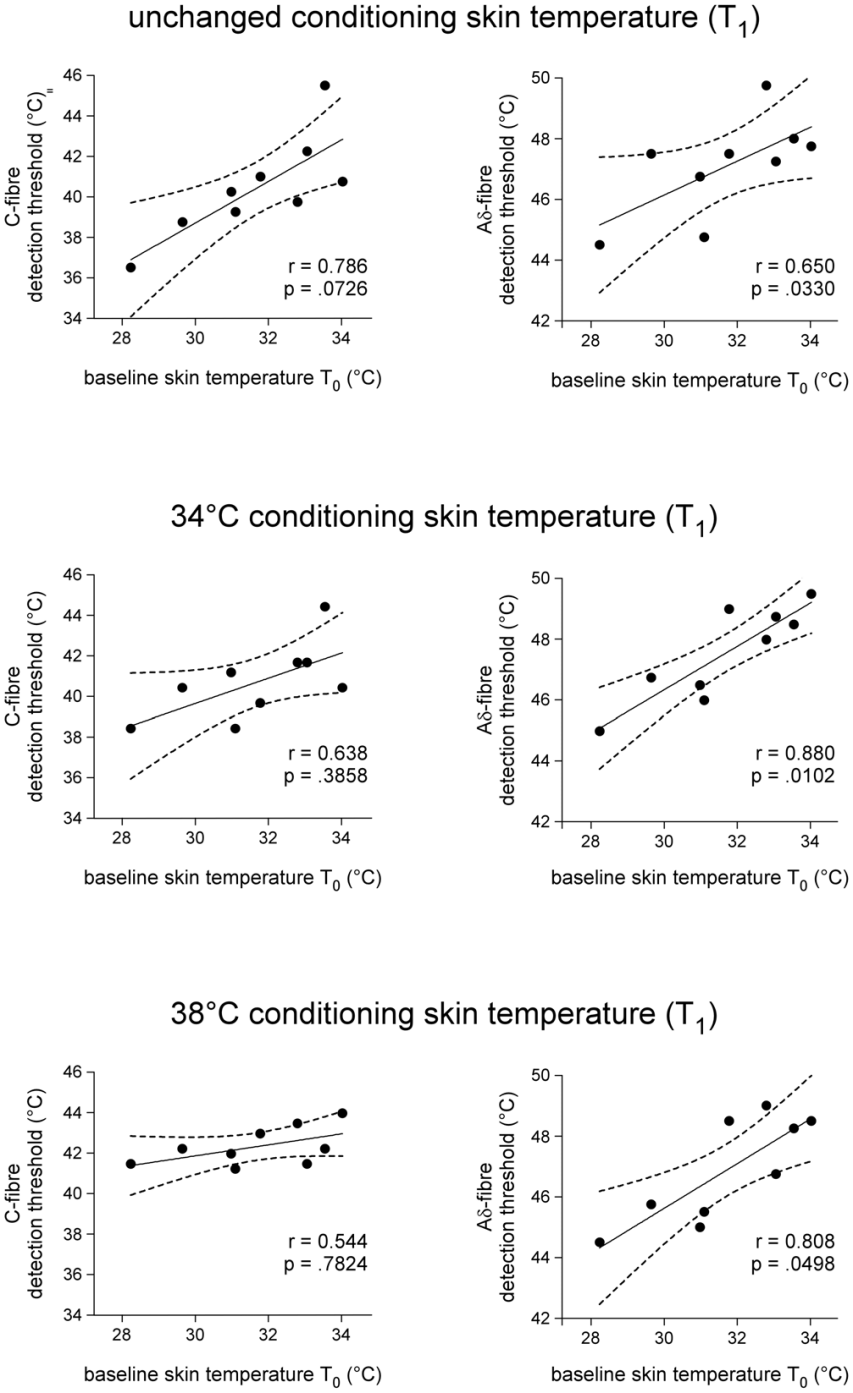
Correlation between baseline skin temperature (T0) measured immediately before trial onset and the thermal detection threshold of the behavioural responses triggered by Aδ- and C-fibre input, in each of the three experimental conditions (T_1_ = unchanged, T_1_ = 34°C and T_1_ = 38°C) (Pearson's correlation coefficient, two-tailed p-values, adjusted for six comparisons using the Bonferroni correction). Note the positive relationship between baseline skin temperature (T_0_) and the Aδ-fibre threshold, in particular, when the stimulated area was brought for two seconds to T_1_ = 34°C and T_1_ = 38°C prior to applying the test stimulus.

Finally, it was examined whether Aδ- and C-thresholds were modulated by the baseline skin temperature (T_0_), i.e. the temperature of the skin before bringing the stimulated area to the conditioning skin temperature (T_1_), also corresponding to the temperature of the skin surrounding the stimulated area during the entire trial duration. For each condition (T_1_ unchanged, 34°C or 38°C), the correlation between baseline skin temperature (T_0_) and the estimated Aδ- and C-threshold temperature (T_2_) was computed using the Pearson correlation coefficient. Results are reported in Figure 5.

## Discussion

In the present study, we show that when brief (50 ms duration) and focal (6-mm beam diameter) CO_2_ laser stimuli are applied to the distal end of a limb, such as to maximize the difference between the peripheral conduction times of unmyelinated C-fibres and myelinated Aδ-fibres, reaction-time latencies can be used to effectively discriminate between behavioural responses to C-fibre input and behavioural responses to Aδ-fibre input. Most importantly, we show that it is possible to obtain, at individual level, a reliable estimate of the thermal detection threshold of Aδ- and C-fibre heat-sensitive afferents, using a novel feedback-controlled CO_2_ laser stimulator to deliver constant-temperature thermal stimuli, combined with an original staircase algorithm relying on reaction-times to distinguish between Aδ-fibre and C-fibre responses. In such, the availability of such a simple behavioural measure of Aδ- and C-fiber function will be of interest to clinicians and researchers aiming at characterizing the physiology and pathophysiology of C-fibres in humans.

Using this approach based on the difference in reaction times to sensory input conveyed by unmyelinated C-fibres and myelinated Aδ-fibres, we confirm that the threshold to obtain time-locked behavioural responses related to the thermal activation of Aδ- and C-fibres by brief and focal infrared laser pulses applied to the hairy skin are significantly different. Indeed, the estimated thermal activation threshold of C-fibres was 39.8±1.7°C, whereas that of Aδ-fibres was 46.9±1.7°C (ΔT = 7.2±1.7°C). The significant difference in the temperature required to elicit Aδ- and C-fibre responses indicates that, provided that the rise in skin temperature generated by the stimulator is controlled as in the present study, brief infrared laser pulses below the threshold of Aδ-fibres but above the threshold of C-fibres can be used to study the behavioural or neural responses related to the selective activation of C-fibre afferents.

### Temperature-controlled CO_2_ laser stimulation of heat-sensitive afferents

The ability to deliver onto the skin radiant heat stimuli that are controlled in temperature offers several significant advantages. Indeed, when it is only possible to define the stimulus in terms of delivered energy, it is very difficult to estimate the actual temperature reached at the level of heat-sensitive free nerve endings, as this is dependent on several, hard to control factors. First, the reflectance, transmission and absorption of the laser energy and, hence, the energy density at the level of heat-sensitive free nerve endings, is dependent on the wavelength of the radiation [24]. Most importantly, at wavelengths <2 µm (e.g. Nd-YAP or Nd:YAG lasers), skin absorption is strongly dependent on skin pigmentation and, therefore, slight variations in skin pigmentation can strongly influence the temperature profile of the delivered stimulus. Second, small variations in the angle of incidence of the beam can result in significant variations in the energy density of the delivered stimulus. Third, even if all the different parameters determining heat transfer to the skin are well controlled, the baseline skin temperature will determine the temperature of the heated skin. These different factors markedly reduce the reproducibility of the stimuli and, thereby, strongly limit the possibilities of exploring the response properties of heat-sensitive afferents.

### C-fibre thermal detection threshold

When the baseline skin temperature was unchanged, the C-fibre threshold was 39.8±1.7°C. Increasing the temperature of the stimulated area to 34°C or 38°C for a short duration before applying the test stimulus led to a significant increase of the C-fibre threshold (Figure 4). One likely explanation to this observation is that increasing the skin temperature before applying the stimulus activates a number of low-threshold heat-sensitive C-fibre afferents, such as C-warm receptors that have been shown to respond to small increases in skin temperature within a range of 30–50°C [25]. This background C-fibre activity, by reducing the contrast of the C-fibre input generated by the test stimulus, could explain why higher stimulus temperatures were required for participants to reliably detect the test stimulus in these two conditions. In fact, it is likely that the behavioural responses triggered by C-fibre input observed in the present study were themselves related mainly to the activation of C-warm receptors, rather than to the activation of heat-sensitive C-fibre *nociceptors*. Indeed, such as what was observed in the present study, C-warm receptors are considered to respond preferentially to sudden increases in skin temperature relative to baseline and, hence, their thresholds are dependent on the background skin temperature [26]–[29]. Furthermore, these studies showed that C-warm receptors respond to such increases in skin temperature in a very phasic manner, compatible with the triggering of time-locked responses such as reaction times. Finally, the C-fibre related detection thresholds observed in the present study were lower than the usually-reported thresholds of C-fibre nociceptors, which are considered to respond only to skin temperatures around and above 45°C [30].

### Aδ-fibre thermal detection threshold

When the baseline skin temperature was unchanged, the Aδ-fibre threshold was 46.9±1.7°C. In contrast to what was observed for C-fibres, increasing the temperature of the stimulated area to 34°C or 38°C immediately before applying the test stimulus did not modulate the Aδ-threshold (Figure 4, right panel). This suggests that the increase in background C-fibre input engendered by the increase in baseline skin temperature did not interfere significantly with the detection of Aδ-fibre input. This could be due to the fact that the pricking sensation generated by Aδ-fibre input is qualitatively very distinct from the sensation elicited by C-fibre input [11] and, hence, easier to discriminate qualitatively from background C-fibre input.

In contrast to the absence of effect of briefly increasing the skin temperature prior to applying the test stimulus (T_1_), we did observe a very significant positive relationship between the Aδ-threshold and T_0_, i.e. the skin temperature before the onset of the trial, also reflecting the temperature of the skin surrounding the stimulus during the entire trial duration (Figure 5, right panels). This indicates that the Aδ-nociceptors triggering the behavioural responses observed in the present study cannot be considered as pure level detectors, i.e. as receptors responding when a given skin temperature is reached regardless of the preceding and/or surrounding skin temperature but, instead, at least partially act as detectors of a change in skin temperature (change relative to the preceding skin temperature, change relative to the surrounding skin temperature). This observation is clearly at variance with the observations of previous studies [31], [32] showing that the threshold for pricking pain is largely invariant with regard to the baseline skin temperature (T_0_). This discrepancy may result from a difference in stimulus parameters. For example, as compared to the 10-ms rise time used in the present study, the rise time of the temperature steps used in these previous studies was much lower (e.g., 3-s in [33]). Interestingly, the correlation between T_0_ and the Aδ-fibre threshold was present even when the temperature of the stimulated area was briefly brought to T_1_ = 34°C or T_1_ = 38°C before applying the stimulus, thus suggesting that the Aδ-threshold was dependent on the skin temperature surrounding the stimulated area rather than the skin temperature of the stimulated area immediately preceding the stimulus. Assuming that Aδ-nociceptors are entirely unresponsive at baseline skin temperatures (T_0_), one must postulate that the threshold for Aδ-fibre input to trigger a behavioural response is not only dependent on the activity conveyed by Aδ-fibres but, instead, that it also integrates other sources of input such as the background C-fibre input whose firing rate is dependent on the baseline skin temperature. In fact, previous studies have also suggested that the perception induced by heating the skin results from a central integration, at spinal and/or cortical level, of the activity of the different classes of heat-sensitive free nerve endings [34]. Further studies are needed to better understand the effect of spatial and temporal summation on the perception of nociceptive input.

Using single-fibre recordings in monkeys, Treede et al. [35] described two distinct classes of heat-sensitive Aδ-fibre polymodal nociceptors, referred to as slowly adapting (type I) and rapidly adapting (type II). Because the response profiles of type I Aδ-nociceptors is not sufficiently phasic, it is usually considered that heat-evoked time-locked responses related to the activation of Aδ-fibres, such as reaction times or event-related potentials triggered by brief laser pulses, are exclusively related to the activation of rapidly-adapting type II Aδ-nociceptors. Compatible with this view, the Aδ-thresholds estimated in the present study matched more closely the thermal activation threshold of type II Aδ-nociceptors (46°C), as compared to type I Aδ-nociceptors (>53°C) [35].

### Conclusion

Using a novel CO_2_ laser stimulator able to deliver constant-temperature heat pulses through a regulated feedback-control of laser power, combined with an original adaptive staircase algorithm using reaction-times to distinguish between responses related to Aδ- and C-fibres, we show that it is possible to estimate reliably the thermal detection thresholds of Aδ- and C-fibres, respectively. Furthermore, we show that it is possible to activate C-fibres selectively and reliably, using a stimulus temperature above the C-fibre threshold, but below the Aδ-fibre threshold, provided that skin temperature is well controlled.

It is important to emphasize that the time-locked behavioural responses to very transient thermal stimuli explored in the present study only reflect the activity of a small subset of the entire population of heat-sensitive free nerve endings located in the epidermis. Indeed, such responses can only be mediated by afferents that are not only heat sensitive but also (1) respond in a phasic manner and (2) elicit a conscious percept. The low threshold of the responses triggered by C-fibre input, as well as the dependency of this threshold on the skin temperature immediately preceding stimulus onset, suggests that C-fibre responses were related mainly to the activation of C-warm receptors. In contrast, the higher threshold of the responses triggered by Aδ-fibre input suggests that these responses were related mainly to the activation of rapidly-adapting type II Aδ-nociceptors.

Taken together, our study provides the basis for a useful and reliable tool to assess the function of unmyelinated C-fibres, which could be very useful in a clinical setting, in particular, for the diagnosis of small fibre neuropathies.
